# 5-Amino-1-β-D-Ribofuranosyl-Imidazole-4-Carboxamide (AICAR) Reduces Peripheral Inflammation by Macrophage Phenotype Shift

**DOI:** 10.3390/ijms20133255

**Published:** 2019-07-02

**Authors:** Lisa Maria Martin, Moritz Möller, Ulrike Weiss, Otto Quintus Russe, Klaus Scholich, Sandra Pierre, Gerd Geisslinger, Ellen Niederberger

**Affiliations:** pharmazentrum frankfurt/ZAFES, Institut für Klinische Pharmakologie, Klinikum der Goethe-Universität Frankfurt, Theodor Stern Kai 7, 60590 Frankfurt am Main, Germany

**Keywords:** AMPK, AICAR, metformin, inflammation, macrophage, cytokines

## Abstract

The stimulation of the AMP-activated kinase (AMPK) by 5-amino-1-β-D-ribofuranosyl-imidazole-4-carboxamide (AICAR) has been associated with antihyperalgesia and the inhibition of nociceptive signaling in the spinal cord in models of paw inflammation. The attenuated nociception comes along with a strongly reduced paw edema, indicating that peripheral antiinflammatory mechanisms contribute to antinociception. In this study, we investigated the impact of AICAR on the immune cell composition in inflamed paws, as well as the regulation of inflammatory and resolving markers in macrophages. By using fluorescence-activated cell sorting (FACS) analysis and immunofluorescence, we found a significantly increased fraction of proresolving M2 macrophages and anti-inflammatory interleukin (IL)-10 in inflamed tissue, while M1 macrophages and proinflammatory cytokines such as IL-1 were decreased by AICAR in wild type mice. In AMPKα2 knock-out mice, the M2 polarization of macrophages in the paw was missing. The results were supported by experiments in primary macrophage cultures which also showed a shift to a proresolving phenotype with decreased levels of proinflammatory mediators and increased levels of antiinflammatory mediators. However, in the cell cultures, we did not observe differences between the AMPKα2+/+ and −/− cells, thus indicating that the AICAR-induced effects are at least partially AMPK-independent. In summary, our results indicate that AICAR has potent antiinflammatory and proresolving properties in inflammation which are contributing to a reduction of inflammatory edema and antinociception.

## 1. Introduction

The AMP-activated kinase (AMPK) is a heterotrimeric protein consisting of two regulatory (β and γ) and one catalytic (α) subunits which all exist in at least two isoforms. The kinase is expressed in several tissues (e.g., liver, heart, skeletal muscle, and brain) and constitutes a metabolic “energy-sensor“ which is activated in states of adenosine-triphosphate (ATP) deficit, e.g., after heat stress, excessive training, hypoxia/ischemia, and starving [1]. Its activation has been associated with the induction of catabolic, energy producing processes such as glucose uptake and fatty acid oxidation [2,3,4,5,6,7], the regulation of appetite, and antiinflammatory [8,9] and antinociceptive effects [10,11]. Therefore, it has been discussed as a useful tool for the treatment of obesity and diabetes [1,12,13], as well as inflammatory diseases and pain [10,11]. Pharmacological AMPK activation with AMPK activators such 5-amino-1-β-d-ribofuranosyl-imidazole-4-carboxamide (AICAR), metformin, or resveratrol as well as exercise-induced AMPK activation leads to a reduction of inflammatory nociception, neuropathic pain, and cancer pain, respectively [14,15,16,17]. In the model of zymosan-induced paw inflammation, our group could show that AMPK activation by AICAR or metformin reduced inflammatory hyperalgesia. In contrast, a complete deletion of AMPKα2, as well as the conditional knock-out of AMPKα2 in immune cells and sensory neurons, resulted in an increased nociceptive response. These results suggest an AMPK-specific effect which is important in immune and neuronal cells in inflammatory nociception. In addition to the decreased nociceptive behavior, AICAR-treated mice revealed a significantly reduced development of zymosan-evoked paw edema in comparison to control mice [14], indicating a strongly suppressed inflammatory response in the periphery. We hypothesized that peripheral effects on immune cells in the edema contribute to the antiinflammatory and antinociceptive effects of AICAR. AMPK activation has already been associated with the resolution of inflammation by the induction of a macrophage phenotype switch towards M2 [18,19]. While proinflammatory M1 macrophages produce proinflammatory mediators, such as interleukin (IL)-1β and tumor necrosis factor (TNF)α, antiinflammatory M2 macrophages produce antiinflammatory factors, such as arginase 1 and IL-10 [20]. The current study aimed to elucidate the effect of AICAR on the inflammatory response in the paw, with a particular focus on the macrophage phenotype and the amount of proinflammatory and proresolving mediators. Furthermore, the AMPK dependence of AICAR-induced effects should be clarified in this context. Therefore, we investigated if a systemic injection of AICAR alters immune cell composition, as well as the level of inflammatory cytokines in the paw of wild type and AMPK knock-out mice in zymosan-induced inflammation. Furthermore, the potential phenotype switch was investigated in mouse macrophages after stimulation with proinflammatory lipopolysaccharide (LPS) and antiinflammatory IL-4—with and without addition of the AMPK activators AICAR and metformin.

## 2. Results 

### 2.1. AICAR Reduces Hyperalgesia and Edema Size in Zymosan-Induced Paw Inflammation

**Figure 1:**
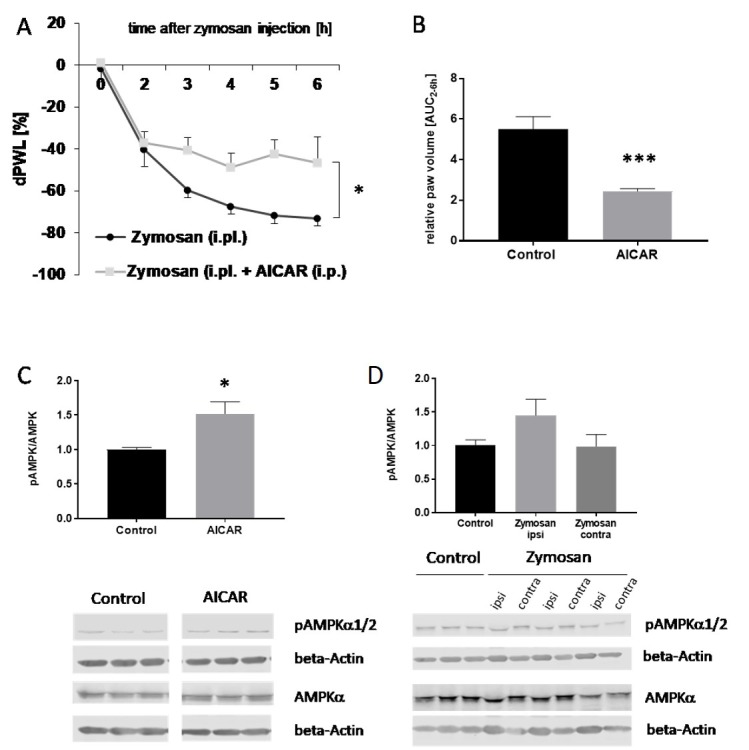
Effects of 5-amino-1-β-D-ribofuranosyl-imidazole-4-carboxamide (AICAR) on AMP-activated kinase (AMPK) activation, mechanical hyperalgesia, and paw edema in zymosan-induced paw inflammation. (**A**) The time course of mechanical hyperalgesia in wild type mice after injection of 10 mg/mL (20 µL) zymosan A into the hind paw with and without addition of AICAR (400 mg/kg i.p.). Zymosan was injected at time ‘0,’ and the response to mechanical stimulation was assessed hourly from 2 to 6 h with a dynamic plantar aesthesiometer. Delta paw withdrawal latency (dPWL) is the relative paw withdrawal latency expressed as a percentage difference between the treated (left) and untreated (right) hindpaws, calculated by the formula: (Left-right)/right × 100. (*n* = 7, zymosan control; *n* = 8, zymosan + AICAR). Repeated measures ANOVA, * *p* = 0.0456, F(1,6) = 6.325. (**B**) Area under the paw volume versus time curve (AUC) from 2 to 6 h after zymosan A injection with and without treatment with AICAR (400 mg/kg body weight, i.p.), (*n* ≥ 6/group). The paw volume was determined by plethysmometric analysis. *** *p* < 0.001 statistically significant difference in comparison with control. (**C**) The densitometric analysis of AMPKα1/2 phosphorylation in paws of mice with (grey column) and without AICAR treatment (black column) as assessed by western blot (*n* ≥ 3/group), independent sample *t*-test,* *p* < 0.05 statistically significant difference in comparison with control. (**D**) The densitometric analysis of AMPKα1/2 phosphorylation in western blots with paws of control mice and ipsi- and contralateral paws of mice treated with zymosan (*n* ≥ 3/group). Phosphorylated (p)-AMPK and AMPK protein levels were normalized with beta-actin, which served as loading control. Then, a ratio between pAMPK and AMPK was calculated.

As a control experiment, we first confirmed results from a previous study [14] using the zymosan-induced paw inflammation model in C57BL/6 mice (Figure 1). As expected, AICAR administration (400 mg/kg body weight (BW) intraperitoneally (i.p.)) led to a significant reduction of the nociceptive behavior in comparison to the control group (Figure 1A). Furthermore, the administration of AICAR significantly reduced the size of zymosan-induced paw edema in comparison to vehicle-treated control mice, indicating that the inflammatory response is suppressed (Figure 1B). To confirm that the i.p. injection of AICAR causes AMPK activation in the paw, the level of phosphorylated (p) AMPK in the paw was assessed by western blot analyses. The results showed a significant increase in p-AMPK in the paw tissue of AICAR-treated mice, thus indicating kinase activation (Figure 1C). The effects of zymosan on AMPK activation were also examined. The inflammatory stimulation induced a slight increase in p-AMPK, which, however, was not significant (Figure 1D). 

The decrease in the nociceptive response and, particularly, the massive reduction of the inflammatory edema led us to the hypothesis that AICAR modulates the immune response in the paw and thereby influences the resolution of inflammation.

### 2.2. AICAR Induces a Macrophage Phenotype Switch in the Inflamed Paw

**Figure 2:**
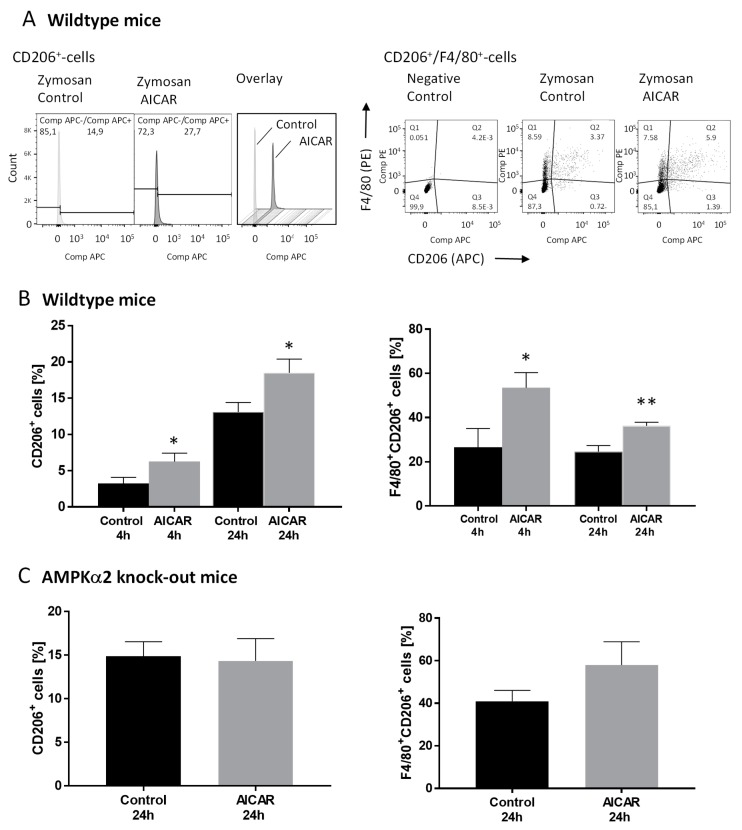
The fluorescence-activated cell sorting (FACS) analysis of CD206-positive immune cells in the zymosan-induced edema. The FACS analysis of CD206^+^ and CD206^+^/F4/80^+^ cells in the paws of mice in the zymosan-induced paw inflammation model. Paw tissue samples were collected 4 (*n* = 3–4/group) and 24 h (*n* = 9/group) after zymosan injection with and without the intraperitoneal injection of AICAR (400 mg/kg body weight). (**A**) Left: Exemplary histograms showing the percentage of CD206^+^ cells in paws of control mice and AICAR-treated mice 24 h after zymosan injection. Right: Exemplary dot blots showing the percentage of CD206^+^/F4/80^+^ cells in paws of control mice and AICAR-treated mice 24 h after zymosan injection. The diagrams in (**B**) show the percentage of CD206 positive cells compared to the total cell count and the percentages of F4/80^+^/CD206^+^ cells in wild type mice 4 and 24 h after zymosan injection. * *p* < 0.05, ** *p* < 0.01 statistically significant difference in comparison with control. (**C**) Percentage of CD206^+^ and CD206^+^/F4/80^+^ cells in the paws of AMPKα2^−/−^ mice 24 h after induction of paw inflammation by zymosan. AICAR-treated mice were compared with control mice. (*n* = 3–4/group).

To further investigate the effects of AICAR on the inflammatory process in the zymosan-injected paw, paws were collected from mice treated with either zymosan alone or in combination with AICAR (400 mg/kg BW i.p.). Immune cells isolated from the edema were stained with markers for macrophages (F4/80^+^) and granulocytes (Ly-6G). To distinguish resident and monocyte-derived macrophages, we stained for Ly-6C. To differentiate between pro-inflammatory (M1) and antiinflammatory (M2) macrophages, staining for CD86 and CD206, respectively, was used and analyzed by fluorescence-activated cell sorting (FACS) (Figure 2, Figure S1). The percentage of macrophages, granulocytes, and monocytes in the paw edema was similar in control and AICAR-treated mice both 4 and 24 h after zymosan injection. Furthermore, AICAR did not influence the ratio between Ly-6G- and Ly-6C-positive macrophages (F4/80^+^Ly-6G^+^, F4/80^+^Ly-6C^+^) and did not alter the number of proinflammatory M1 macrophages (CD86^+^ and F4/80^+^CD86^+^) (Figure S1A). In contrast, AICAR increased the number of antiinflammatory M2 macrophages (CD206^+^, F4/80^+^CD206^+^) significantly after both 4 and 24 h (Figure 2B). To investigate if this effect is specifically mediated via AMPK activation, a FACS analysis was also performed using AMPKα2^−/−^ mice. The total cell count of macrophages, granulocytes, and monocytes after zymosan injection was comparable with wild type mice and did not show the effects of AICAR treatment. This was also the case for Ly-6G and Ly-6C positive macrophages (Figure S1B). However, in comparison to wildtype mice, AICAR treatment led to a slight but not significant decrease in CD86^+^ (but not F4/80^+^CD86^+^) cells and did not increase the percentage of F4/80^+^CD206^+^ cells (Figure 2C), thus indicating that the AICAR-induced shift to a proresolving inflammatory state was mediated via AMPK.

**Figure 3:**
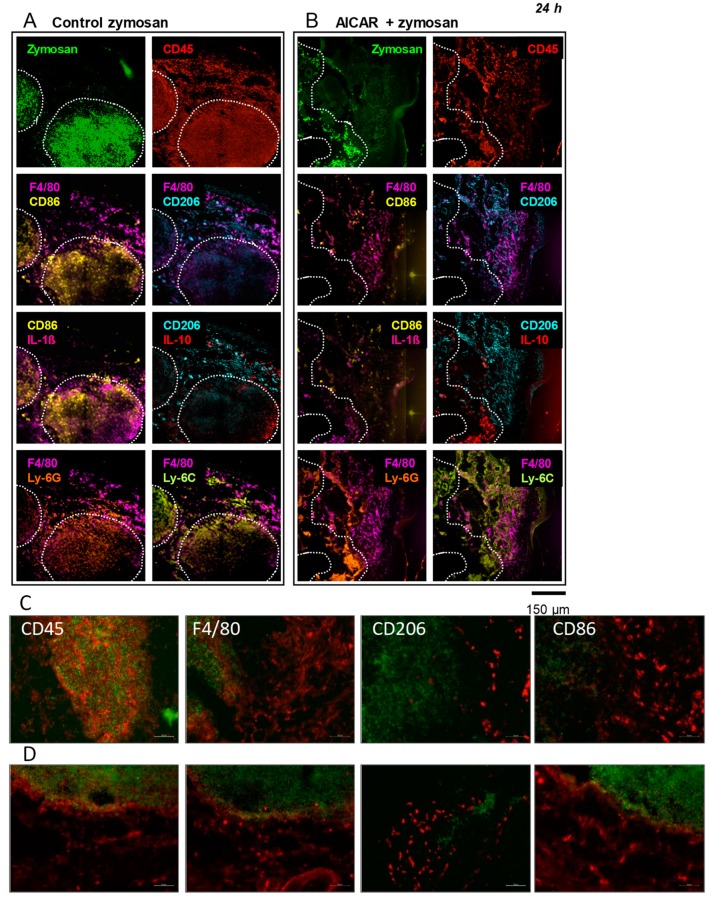
Immunofluorescence of immune cells and inflammatory markers in the inflamed paw. (**A**,**B**) Representative multi epitope ligand cartography (MELC) immunostainings of macrophage markers in the paw of mice 24 h after the injection of zymosan (**A**) or zymosan + AICAR (**B**). The dotted white lines indicate the outline of the area containing FITC-zymosan (green). The respective images show CD45 staining (red) as well as the co-stainings of F4-80 (pink) with CD86 (yellow), CD206 (turquoise), Ly-6G (orange), and Ly-6C (light green), respectively. Furthermore, co-stainings are shown for CD86 with interleukin (IL)-1β (pink) and CD206 with IL-10 (red).The pictures show representative data of three independent experiments. The images are shown in false colors. (**C**,**D**) are the representative stainings of three independent immunofluorescence experiments with zymosan (**C**) and zymosan + AICAR-treated mice (**D**) to confirm the data from the MELC analysis. The green staining shows the zymosan-infiltrated areas, and red staining indicates CD45, F4/80, CD206, and CD86 positive cells. Scale Bar: 50 µm.

Multi epitope ligand cartography (MELC) was performed using paw tissue sections 4 and 24 h after the intraplantar injection of Fluorescein isothiocyanate (FITC)-zymosan (20 μL, 10 mg/mL) with and without AICAR treatment (Figure 3 and Figure S2). The method allows for the visualization of several different markers on the same slide and, thereby, the analysis of the impact of AMPK activation on the expression and localization of immune cell phenotypes and the expression of inflammatory mediators in inflamed tissue. 24 h after zymosan injection, there was a slight decrease of leukocyte (CD45) and macrophage (F4/80^+^) accumulation in the zymosan-injected area in AICAR-treated mice in comparison to control mice. Furthermore, in AICAR-treated mice, there were less inflammatory (M1) macrophages (CD86) in the paw tissue as compared to the control, where the entire zymosan-covered area was infiltrated by CD86 positive cells. In accordance with the FACS analysis, antiinflammatory (M2) macrophages (CD206) accumulated in the inflamed paw tissue of AICAR-treated mice in comparison with control animals. The number of IL-1β positive cells was strongly decreased by AICAR treatment in comparison with the control paw tissue, where they were highly stained in the zymosan-injected area. IL-1ß staining was detected in the same areas as observed for proinflammatory macrophages (CD86), supporting its release by these cells. Furthermore, the number of IL-10 positive cells was increased in the AICAR condition in the same region that was positive for CD206 positive macrophages. Regarding granulocytes (Ly-6G) and blood-derived leukocytes (Ly-6C), no distinct differences could be observed. In both conditions, the two antigens were mostly co-localized, covering both the zymosan-injected area and the tissue surrounding it (Figure 3A,B). Similar results were observed 4 h after zymosan injection, except for the AICAR-induced decrease of CD86 positive cells, which seems to be regulated at later time points (Figure S2A,B). To confirm the results from the MELC analyses, we additionally performed immunofluorescence for the analysis of immune cell markers. In zymosan-treated mice, we observed a high number of CD45-positive leukocytes as well as F4/80- and CD86-positive macrophages in the paw sections. Similar to the MELC analyses, the number of CD45 and F4/80 positive cells was reduced in AICAR-treated mice. Proinflammatory (CD86-positive) macrophages were also decreased, while antiinflammatory (CD206-positive) macrophage invasion was strongly enhanced after AICAR treatment in comparison to zymosan-treated controls (Figure 3C,D).

### 2.3. AICAR Reduces Proinflammatory Markers and Induces Resolution Markers in Macrophages

**Figure 4:**
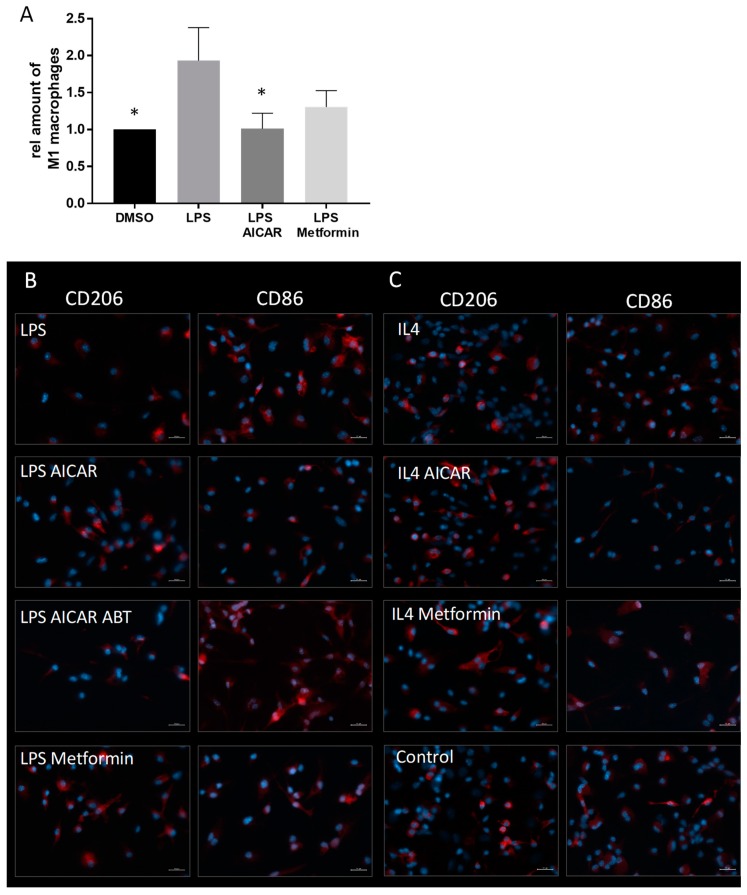
Effects of AICAR and metformin on the phenotype of primary bone marrow derived macrophages. (**A**) FACS analysis showing a lipopolysaccharide (LPS)-induced shift towards a M1 phenotype indicated by an increase of CD86 positive cells. This phenotype switch is significantly inhibited by AICAR. (*n* = 3). * *p* < 0.05, (**B**) Immunofluorescence showing CD86 and CD206 positive cells (both shown in red) in differentially treated bone marrow-derived macrophage (BMM) cultures. The cells were either incubated with vehicle, proinflammatory LPS, or antiinflammatory IL-4. Furthermore, the cells were co-incubated with LPS and AICAR, AICAR and adenosine kinase inhibitor ABT702, as well as metformin. IL-4-treated cells were also co-incubated with AICAR and metformin. The pictures show a representative analysis of three independent incubations. The cellular nuclei are visualized by DNA staining with 4′,6-Diamidin-2-phenylindol (DAPI) (blue). Scale Bar: 20 µm.

To investigate the influence of AMPK activators on the phenotype of cultured macrophages, we prepared primary bone marrow-derived macrophage (BMM) cultures from mice (Figure 4). To polarize the macrophages towards a pro- or antiinflammatory phenotype, we treated the cultures either with the proinflammatory TLR-4 agonist lipopolysaccharide (LPS) or the antiinflammatory cytokine interleukin 4 (IL-4) with and without addition of the AMPK activators AICAR and metformin. In BMM, LPS treatment induced a strong increase of CD86 positive cells, as assessed by FACS analysis (Figure 4A) and immunofluorescence. This LPS-induced increase was inhibited by the addition of AICAR and metformin. In contrast, immunofluorescence showed a low expression of CD206 in LPS-treated controls, which was strongly augmented in the AICAR- and metformin-treated cultures (Figure 4B). IL-4 stimulated macrophages revealed a low expression of CD86 and a relatively high expression of CD206, both of which remained stable by treatment with AICAR and metformin, respectively (Figure 4C). These experiments suggest a shift towards a proresolving M2 phenotype of the BMMs. AICAR-induced AMPK activation is mediated by its phosphorylation by adenosine kinase to form 5-amino-1-β-D-ribofuranosyl-imidazole-4-carboxamide monophosphate (ZMP), its active metabolite. The addition of ABT702, an adenosine kinase inhibitor which suppresses the conversion of AICAR to ZMP, failed to inhibit the LPS-induced CD86 increase observed after AICAR treatment, thus indicating the effect was AMPK-specific (Figure 4B, Figure S3). 

**Figure 5:**
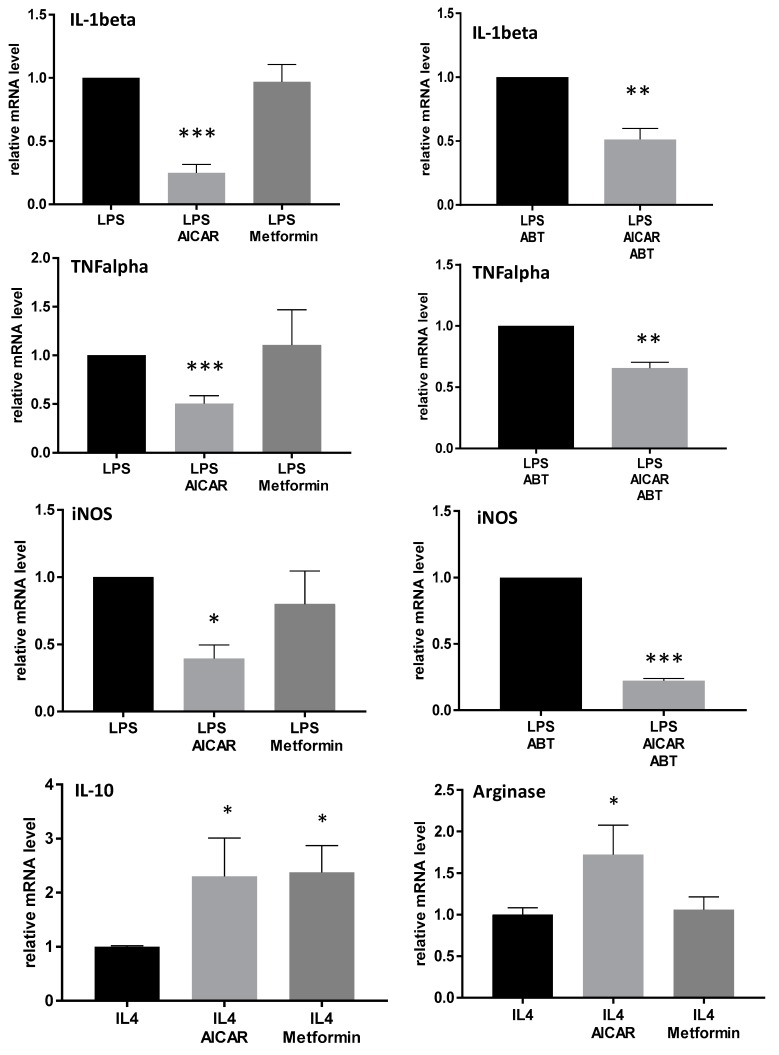
Effects of AICAR and metformin on the expression of inflammatory genes in primary bone marrow-derived macrophages. qRT-PCR analyses of proinflammatory IL-1beta, TNF alpha, iNOS, and antiinflammatory IL-10 and arginase 1 in LPS- and IL-4-stimulated BMMs with and without treatment with AICAR [500 µM] or metformin [500 µM], or AICAR in combination with its activation inhibitor ABT702, respectively. Signals for specific genes were normalized with glyceraldehyde 3-phosphate dehydrogenase (GAPDH). The relative quantitative level of samples was determined by standard 2^-dd*C*^ calculations and expressed as fold-change compared to a reference control sample. (*n* = 3–4/treatment), * *p* < 0.05, ** *p*< 0.01, *** *p*< 0.001 statistically significant difference in comparison with LPS or IL-4-treated controls.

qRT-PCR analyses revealed an inhibition of LPS-induced IL-1β, TNFα, and inducible nitric oxide sythesis (iNOS) expression in BMM and a further increase of IL-4-induced IL-10 and arginase 1 expression in cells treated with AICAR, in comparison to LPS- and IL-4-stimulated controls. Interestingly, in contrast to the immunofluorescence, metformin did not reduce the LPS-induced increase in inflammatory mediators; it only significantly increased antiinflammatory IL-10. Additional treatment with ABT702 as an inhibitor of AICAR conversion revealed similar effects to those observed with AICAR alone (Figure 5). These data indicate that there might be additional mechanisms of AICAR apart from AMPK activation that contribute to the macrophage phenotype switch. To further assess the role of AMPK in AICAR-induced effects, we performed RT-PCR experiments with macrophages derived from AMPK wild type and knock-out mice. In macrophages from AMPKα2 knock-out mice, the effects were similar to that in wild type mice. IL-1β and TNFα were still reduced after AICAR treatment; however, effects were a little less pronounced. iNOS expression was even more strongly inhibited in AMPK knock-out macrophages (Figure S4). These results further suggest AMPK-independent effects of AICAR in macrophages. Metformin inhibited TNFα and iNOS expression in macrophages derived from AMPKα2 wild type mice but not in knock-out littermates, thus indicating that the metformin-mediated effects are AMPK-specific.

## 3. Discussion

Previous data from our group indicated that the activation of AMPK by AICAR leads to reduced inflammatory hyperalgesia which is associated with potent local antiinflammatory effects in the zymosan-induced paw inflammation model. The antinociceptive effects are mainly mediated via the α2 subunit of the enzyme in sensory neurons and immune cells, thus leading to alterations of pronociceptive signaling pathways in the nervous system [14]. These results were supported by a recent study using the complete Freund’s adjuvant (CFA)-paw inflammation model. In this model, the local administration of AICAR reduced inflammatory nociception and inhibited NF-κB activation and IL-1β expression in the paw [21]. However, the complete antiinflammatory mechanisms of AICAR in the inflamed paw which contribute to antinociceptive effects have not yet been clarified. In this study, we investigated the effects of AICAR on immune cells under inflammatory conditions in vivo and in vitro. In the paw inflammation model, we found that AICAR-induced macrophage polarization towards an antiinflammatory M2 phenotype was associated with an increase of antiinflammatory cytokines and a decrease of proinflammatory mediators in the inflamed paw. Similar results could be detected in primary mouse macrophages after stimulation with LPS or IL-4 in combination with AICAR. 

Several reports showed that AMPK activation is involved in anti-inflammatory effects in cellular, preclinical, and clinical studies, e.g., on diabetes and obesity [9,22]. Macrophage polarization to an antiinflammatory phenotype that is associated with AMPK-activation has already been shown in macrophage cell culture, as well as in disease models and patient samples, e.g., in rheumatoid arthritis or obesity [19,23,24]. Furthermore, AICAR was associated with the macrophage phenotype switch in cells derived from obese mice or patients [25], and in an atherosclerosis model in mice [25,26]. The activation of AMPK by AICAR is mediated by its active metabolite ZMP. ZMP binds to the AMP binding site of the gamma-subunit of the enzyme and thereby causes its activation. Besides AMPK activation, AICAR has been associated with AMPK-independent effects such as the inhibition of cell proliferation and the induction of apoptosis [27,28], as well as macrophage ER stress responses [29]. Therefore, it cannot be excluded that the observed AICAR-induced antiinflammatory effects are at least partially AMPK-independent. We have performed additional experiments using AMPKα2 knock-out mice as well as cells treated with ABT702, an inhibitor of adenosine kinase which suppresses the conversion of AICAR to ZMP. In AMPKα2 knock-out mice, the inflamed tissue derived from the paw edema did not show the increase of CD206 antiinflammatory cells after AICAR treatment, as observed in wild type mice; this indicates that AICAR-induced effects are AMPK-specific. This assumption is supported by studies showing the AMPK-dependent regulation of M1 and M2 genes in macrophages from patients with rheumatoid arthritis and M2 polarization during muscle regeneration in mice [18,23]. Furthermore, AMPK is directly involved in the macrophage polarization to an antiiflammatory phenotype in murine and human primary macrophages [19]. On the other hand, in primary bone marrow derived macrophages, ABT702 only partially reversed AICAR-induced effects, and AMPKα2 knock-out macrophages showed similar effects as wild type cells. Thus, there are at least some additional AMPK-independent actions of AICAR in macrophages which have already been described. In accordance with our data, the AICAR mediated inhibition of LPS-induced iNOS expression in macrophages and microglia cells has been described as AMPK-independent [30]. This effect might be at least partially due to the AMPK-independent inhibition of NF-κB/RNA PolII and signal transducer and activator of transcription (STAT)3 transcription factor activation which has been observed in primary human macrophages [31]. Furthermore, it has been described that AICAR promotes efferocytosis, an important mechanism of macrophages in resolving inflammation, in an AMPK-independent manner [32]. In addition, it has to be noted that AMPK-induced effects in macrophages were often associated with the α1 isoform of the enzyme, and, therefore, the missing effect in AMPKα2 macrophage cultures might be due to the fact that this is not the primary subunit in these isolated cells [19,33] but appears to be important for macrophages in the paw edema. 

The mechanism of metformin-induced AMP-activation is not completely elucidated yet. It has been suggested to activate AMPK in an indirect manner [34], possibly via the complex I of the mitochondrial respiratory chain [35,36] and the modification of the intracellular AMP:ATP ratio. In our experiments, metformin induced a reduction of proinflammatory markers in AMPKα wild type BMMs which was not altered by an AMPKα2 knock-out, thus indicating that metformin effects might be more specifically mediated via AMPK than AICAR-induced effects. Metformin has previously been described as an AMPK-dependent inductor of macrophage polarization [24,37]. In those cases, the drug concentration for metformin was higher than in our study (e.g., 2 mM or 5 mM vs 500 µM in this study), which might explain the missing effect in our experiments using BMMs from C57BL/6 mice.

Taken together, our results clearly show that AICAR has potent antiinflammatory properties in painful inflammation which are due to a macrophage polarization toward an antiinflammatory M2 phenotype. This effect reduces the expression of proinflammatory mediators while increasing antiinflammatory mediators. In conclusion, we could show that the AMPKα2 subunit contributes to AICAR-induced macrophage polarization directly in the inflamed area of the paw. In isolated BMMs, it is likely that different subunits and AMPK-independent mechanisms are also involved in the AICAR-induced modulations. Nevertheless, our data support preclinical and clinical studies showing the anti-inflammatory and antinociceptive potential of AMPK-activating drugs [38,39].

## 4. Materials and Methods

### 4.1. Materials

#### 4.1.1. Animals 

Ethics Statement: In all animal experiments, the ethical guidelines for investigations in conscious animals were obeyed, and the procedures were approved by the local Ethics Committee for Animal Research (Regierungspräsidium Darmstadt, Germany, permit no. F95/53 (approved 9 September 2017), FK/1115 (approved 8 April 2019)). All efforts were made to minimize animal suffering and to reduce the number of animals used.

Male C57BL/6 mice were obtained from Charles River, Sulzfeld, Germany, at the age of 6–8 weeks. Homozygous AMPKα2^−/−^-mice with a C57BL/6 background were kindly provided by Professor Benouit Viollet, France. These mice lack exon C of the AMPK gene which encodes the catalytical subunit of AMPKα2 and results in kinase inactivation. AMPKα2^−/−^ mice are viable, fertile, showed no apparent differences to their wild type littermates, and have a normal life span [40]. For behavioral experiments, AMPKα2^-/-^-mice were backcrossed with C57BL/6 wild type mice. Heterozygous offspring were then further bred to gain wild type and AMPKα2^−/−^ littermates. Genotyping was performed using the following primers as described below: 


***AMPK Genotyping***
657  5′-GCTTAGCACGTTACCCTGGATGG-3′658  5′-GTTATCAGCCCAACTAATTACAC-3′659  5′-GCATTGAACCACAGTCCTTCCTC-3′

The genotyping primers were used in 2 combinations. The combination of 657 and 658 detects the AMPKα2 wild type and generates a product of ~200 bp. The combination of 657 and 659 is used for genotyping of knock-out and generates a product of ~700 bp. Annealing was performed at 58 °C for 90 s, and elongation was performed at 72 °C for 90 s in 35 cycles. Animals had free access to food and water and were maintained in climate- and light-controlled rooms (24 ± 0.5 °C, 12/12 h dark/light cycle). All behavioral experiments were performed by an observer blinded for the genotype in a dedicated room with restrictions on sound levels and activities.

#### 4.1.2. Reagents

AICAR and metformin used as AMPK activators were purchased from Merck Chemicals Ltd. (Darmstadt, Germany). For animal experiments, AICAR was dissolved in 0.9% NaCl at a concentration of 50 mg/mL. C57BL/6 wild type and AMPKα2 knock-out mice received intraperitoneal injections of the AICAR solution corresponding to a dose of 400 mg/kg body weight [13]. Control mice received the same volume of vehicle. For cell culture experiments, stock solutions of AICAR and metformin were prepared in 100% dimethylsulfoxide (DMSO). Zymosan A was purchased from Sigma-Aldrich, Munich, Germany. Antibodies and primers are indicated in the respective method sections.

### 4.2. Methods

#### 4.2.1. Zymosan-Induced Paw Inflammation

Hind paw inflammation in mice was induced by the subcutaneous injection of 20 µl of a 10 mg/mL zymosan A (Sigma-Aldrich, Munich, Germany) suspension in phosphate buffered saline (0.1 M, pH 7.4) into the mid plantar region of the left hind paw [41]. Baseline mechanical sensitivity was determined before zymosan injection using a Dynamic Plantar Test device (Ugo Basile, Varese, Italy). The device consists of a steel rod that is pushed against the plantar surface of the paw with increasing force until the paw is withdrawn. The maximum force was set at 5 g to prevent tissue damage, and the ramp speed was 0.5 g/s. Mice were placed in test cages with a metal grid bottom. They were kept in the test cages for 1 h to allow for accommodation. The paw withdrawal latency (PWL) was obtained as the mean of 6 consecutive assessments at each time point (at least 10 s between repeated measurements of the same paw) starting 1 h after zymosan A injection and then hourly up to 6 h. A second group of animals was used to determine the volume of zymosan-induced paw edema with a plethysmometer (IITC Life Science, Woodland Hills, CA, USA) according to the manufacturer’s instructions. After measuring a baseline, zymosan A was injected, and the paw volume was determined hourly for 6 h. The area under the paw volume versus time curve (AUC) was calculated.

#### 4.2.2. FACS Analysis

Fluorescence activated cell sorting (FACS) was used to investigate the quantity of specific immune cells in the paw tissue after zymosan-induced inflammation. Therefore, mice were sacrificed and the paw edema was collected either 4 or 24 h after zymosan injection and stored in 1× phosphate buffered saline (PBS) on ice. The tissue was cut into small pieces and transferred into a 2 mL safe-lock tube containing 500 μL of a 37 °C warm lysis buffer (Dulbecco’s Modified Eagles Medium (DMEM)) with 3 mg/mL of collagenase A (Sigma, Deisenhofen, Germany); it was then incubated at 37 °C for 45 min. Subsequently, the suspension was filtered through a 70 μm BD Biosciences Falcon Cell Strainer and centrifuged for 5 minutes at 1000× *g* at room temperature. The supernatant was discarded, and the cells were resuspended in a 400 μL erythrocyte lysis buffer (135 mM NH4Cl, 10 mM NaHCO3, 0.1 mM Na-EDTA, pH 7.2) and incubated at room temperature for 5 min. After subsequent centrifugation (5 min, 1000× *g*, RT), the supernatant was discarded, and the cells were resuspended in 120 μL of 1× PBS. The cell solution was transferred into a 96 conical bottom well plate and centrifuged (5 min, 1000× *g*, RT). The supernatant was removed, and the pellet was resuspended in a 90 μL FACS buffer (1× PBS + 1% FCS, 4 °C). The cells were then stained with epitope-specific markers of immune cells using PE-F4/80 (Miltenyi Biotec, Bergisch Gladbach, Germany), APC-Ly-6C (eBioscience, ThermoFisher Scientific, Waltham, MA USA), FITC-Ly-6G (Miltenyi Biotec, Bergisch Gladbach, Germany), FITC-CD86 (Biolegend, San Diego, USA), and APC-CD206 (Biolegend, San Diego, CA, USA) antibodies for 1 hour at room temperature. Cells without the addition of antibodies served as negative controls. The cell pellet was washed 2 times with a 100 μL FACS buffer, followed by centrifugation at 1000× *g* for five minutes. The pellet was resuspended in 100 μL 4% paraformaldehyde (PFA) in PBS and stored at 4 °C until FACS measurement. The FACS measurement was performed using a BD FACS Canto II instrument and the software ‘FACS Diva’ (BD Biosciences, San Jose, CA, USA). Data were analyzed using the ‘FlowJo LLC V10′ software.

#### 4.2.3. Multi-Epitope-Ligand-Cartography (MELC) 

For MELC analysis, mice were injected with 20 μL of FITC-labeled zymosan (10 mg/mL in 1× PBS) (Life Technologies, Darmstadt, Germany) into the plantar surface of the hind paw, with and without additional intraperitoneal injection of AICAR. After either 4 or 24 h, the mice were sacrificed, and the paw edema was collected and directly embedded into cryomolds with optimum cutting temperature (O.C.T.) Compound (Tissue-Tek, Sakura, ThermoFisher Scientific, Waltham, MA USA) using dry ice. The tissue was cryosectioned into 10 μm thick sections at −21 °C and placed on silane-coated coverslips stored at −80 °C until MELC analysis. Coverslips containing FITC-labeled zymosan were identified using a fluorescent microscope (Zeiss Axioimager, Carl Zeiss AG, Oberkochen, Germany). For imaging, the sections were fixed using 4% PFA in PBS for 15 min, followed by permeabilization with 0.1% Triton X-100 in PBS for 15 min and blocking with 3% bovine serum albumin (BSA) in PBS for one hour. MELC analysis was performed on the stage of a wide-field microscope (DM IRE2; Leica, Wetzlar, Germany). A background picture was taken, and then the slides were incubated with different fluorescence-labelled antibodies (see below) by a robotic process for 15 min each. After a washing step in PBS, phase contrast and fluorescence images were captured by a cooled charge-coupled device camera (Apogee KX4; Apogee Instruments, Roseville, CA, USA), and bleaching steps were performed between every antibody incubation. After the staining was completed, all fluorescence images were aligned pixel-wise using the corresponding phase contrast images, and flat-field correction was used to eliminate illumination errors. [42,43]

The following antibodies were used for MELC analyses:

CD45 FITC, (Miltenyi Biotec, Bergisch Gladbach, Germany, 1:200) 

F4/80 PE (Biolegend, San Diego, CA, USA, 1:400) 

CD86 PE (Biolegend, San Diego, CA, USA, 1:200)

CD206 APC (Bio-Rad, CA, USA 1:400) 

Ly-6G PE (eBioscience, Thermo FisherScientific, Waltham, MA, USA, 1:400) 

Ly-6C APC (eBioscience, Thermo FisherScientific, Waltham, MA, USA, 1:200) 

IL-1ß FITC (Thermo FisherScientific, Waltham, MA, USA 1:100) 

IL-10 FITC (eBioscience, Thermo FisherScientific, Waltham, MA, USA 1:50)

#### 4.2.4. Western Blot Analysis

For the western blot analysis of AMPK activation, the contralateral paw of zymosan A injected mice was used. Tissues were homogenized in a PhosphoSafe Extraction Buffer (Merck, Darmstadt, Germany) containing a protease inhibitor (1 mM Pefabloc SC, Alexis Biochemicals, Lausen, Switzerland) immediately after preparation. To remove cellular debris, extracts were centrifuged at 14,000 rpm for 1 h at 4 °C. The supernatants were stored at −80 °C. 

Proteins in tissue homogenates (30 µg) were separated electrophoretically by 10% SDS-PAGE and then transferred onto nitrocellulose membranes by wet-blotting. To confirm equal loading, all blots were stained with Ponceau red solution. Membranes were blocked for 60 min at room temperature in an Odyssey blocking reagent (LI-COR Biosciences, Bad Homburg, Germany) diluted 1:2 in 0.1 M PBS, pH 7.4. Then, the blots were incubated overnight at 4 °C with primary antibodies against p-AMPKα1/2 or AMPK (both 1:250, Cell Signaling Technology, Boston, MA, USA) in a blocking buffer. After washing three times with 0.1% Tween 20 in PBS, the blots were incubated for 60 min with an IRDye 800- or IRDye 700-conjugated secondary antibody (Molecular Probes, 1:10,000 in a blocking buffer). After rinsing in 0.1% Tween 20 in PBS, protein-antibody complexes were detected with the Odyssey Infrared Imaging System (LI-COR, Bad Homburg, Germany). Beta-actin (42 kDa) (Sigma, Deisenhofen, Germany) was used as the loading control. A densitometric analysis of the blots was performed with Image Studio Software (LI-COR, Bad Homburg, Germany).

#### 4.2.5. Cell Culture

Bone marrow derived macrophages (BMM) were generated from C57BL/6, AMPKα2^−/−^, and AMPKα2^+/+^ mice. Animals were killed by CO_2_ and cardiac puncture. Muscle tissue was removed from the hind legs, and the bones were cut directly beyond and above the joints. The bones were placed into a perforated 0.2 mL tube which was inserted into a 1.5 mL reaction tube. After centrifugation at 16,800× *g* for 15 s, the bone marrow was resuspended in a RPMI1640 medium supplemented with 10% FCS, 1% Pen/Strep, and 20 ng/mL recombinant murine macrophage colony stimulating factor (M-CSF, Peprotech, Hamburg, Germany). Cells were seeded in 6 or 12 well plates and cultivated and differentiated for 7 d before stimulation. BMM were stimulated with lipopolysaccharide (0.1 µg/mL) or interleukin (IL)-4 (20 ng/mL) with and without addition of AICAR and metformin (both 500 µM), respectively. Since both drugs were dissolved in DMSO, solvent concentrations were adjusted to 0.5% in all incubations. Cells incubated with 0.5% solvent alone served as controls. After an incubation time of 4 h, cells were subjected to mRNA preparation. 

#### 4.2.6. Quantitative Real-Time PCR 

RNA was prepared from primary bone marrow macrophages using TriReagent, as described previously [44]. 800 ng of total RNA was used for the reverse transcription, which was performed with the Thermo Scientific Verso cDNA system. (Thermo Fisher Scientific GmbH, Waltham, MA USA). 20 ng of an RNA equivalent were subjected to real-time PCR in an Applied Biosystems Sequence Detection System Quant5 using a FastStart Universal Master Mix Kit (ThermoFisher Scientific GmbH, Waltham, MA USA) with SYBR Green fluorescence staining. The mRNA expression of the respective genes was determined and normalized to GAPDH mRNA. The following gene-specific primers were used:

Arginase 1 FW 5’-GTGAAGAACCCACGGTCTGT-3’

Arginase 1 RV 5’-CTGGTTGTCAGGGGAGTGTT-3’

IL-1β FW 5’-CTGGTGTGTGACGTTCCCATTA-3’

IL-1β RV 5’-CCGACAGCACGAGGCTTT-3′

IL-10 FW 5’-GCTCTTACTGACTGGCATGAG-3’

IL-10 RV 5’-CGCAGCTCTAGGAGCATGTG-3’

iNOS FW 5’-CCAAGCCCTCACCTACTTCC-3’

iNOS RV 5’-CTCTGAGGGCTGACACAAGG-3’

TNFα FW 5’-GCTGAGCTCAAACCCTGGTA-3’

TNFα RV 5’-CGGACTCCGCAAAGTCTAAG-3’

GAPDH FW 5’-CAATGTGTCCGGATCT-3’

GAPDH RV 5’-GTCCTCAGTGAAGATG-3’

The cycle number at which the fluorescence signals cross a defined threshold (Ct-value) is proportional to the number of RNA copies present at the start of the PCR. The threshold cycle number for the specific mRNA was standardized by subtracting the Ct-value of GAPDH from the Ct-value of the respective gene of the same sample. The relative quantitative level of samples was determined by standard 2^-ddCt^ calculations and expressed as fold-change compared to a reference control sample.

#### 4.2.7. Immunofluorescence

The immunofluorescence staining of mouse paw tissue sections was performed to confirm the results from MELC. After zymosan- and AICAR-treatment, mice were perfused with 2% PFA in 1× PBS, and the hind paws were collected. The plantar sides of the paws were dissected and placed in 2% PFA in 1× PBS. Afterwards, they were incubated in a 20% sucrose solution for at least five hours and stored in a 30% sucrose solution overnight at 4 °C. The tissues were then embedded in cryomedium (Tissue-Tek, Sakura, ThermoFisher Scientific, Waltham, MA, USA), frozen on dry ice and cut into 12 μm thick cryosections at −21 °C. The slides were stored at 4 °C until needed for histological staining.

For antibody staining, the sections were washed in PBS and then incubated in PBSTx (1× PBS + 0.1% Triton X-100) for 10 min, followed by incubation in a blocking solution (PBSTx + 3% BSA + 10% normal goat serum (NGS)) at room temperature for 60 min. Afterwards, primary antibodies against CD45 (BD Biosciences, USA), F4/80 eBioscience, Thermo Fisher Scientific, Waltham, MA, USA), CD 86, Biolegend, Koblenz, Germany), and CD206 Biolegend, Koblenz, Germany) diluted 1:1000 in PBSTx + 3% BSA were applied, and the sections were stored at 4 °C overnight. After washing with PBSTx for 3× 10 min, the secondary antibody (anti-rat IgG AlexaFluor 488, (Life Technologies, ThermoFisher Scientific, Waltham, MA, USA), 1:800 in PBSTx + 3% BSA) was applied for 2 h at RT. After a final washing step, the sections were fixed with a mounting medium (Aqua Polymount, Polysciences, Hirschberg, Germany) and cover slips. Microscopy was performed using an inverse fluorescence microscope (Zeiss Axioimager, Carl Zeiss AG, Oberkochen, Germany).

#### 4.2.8. Data Analysis

Statistical evaluation was done with GraphPad Prism 7 (GraphPad Software Inc., San Diego, CA, USA). Data are presented as means ± SEM. Data were either compared by a univariate analysis of variance (ANOVA) with subsequent *t*-tests employing a Dunnett’s correction for multiple comparisons or by a Student’s *t*-test. For the analysis of inflammatory hyperalgesia in the zymosan A-induced paw inflammation, paw withdrawal latencies in response to mechanical stimulation were expressed as the relative difference between the zymosan A-treated left and the untreated right hind paw. This was calculated as: ΔPWL = (left-right) / right x 100 (mean ± SEM). For the analysis of the time courses after zymosan injection, a repeated measures ANOVA was performed. The area under the paw volume versus time curve (AUC) was calculated by employing the linear trapezoidal rule. For all tests, a probability value *p* < 0.05 was considered as statistically significant.

## Supplementary Materials

Supplementary materials can be found at https://www.mdpi.com/1422-0067/20/13/3255/s1.
