# Supplementary material for: 5-Amino-1-β-D-Ribofuranosyl-Imidazole-4-Carboxamide (AICAR) Reduces Peripheral Inflammation by Macrophage Phenotype Shift
Source: Int J Mol Sci. 2019 Jul 2;20(13):3255. doi: 10.3390/ijms20133255 (PMC6651813; doi:10.3390/ijms20133255)
Supplement: Supplementary file 1 [file ijms-20-03255-s001.pdf]

Supplementary figures

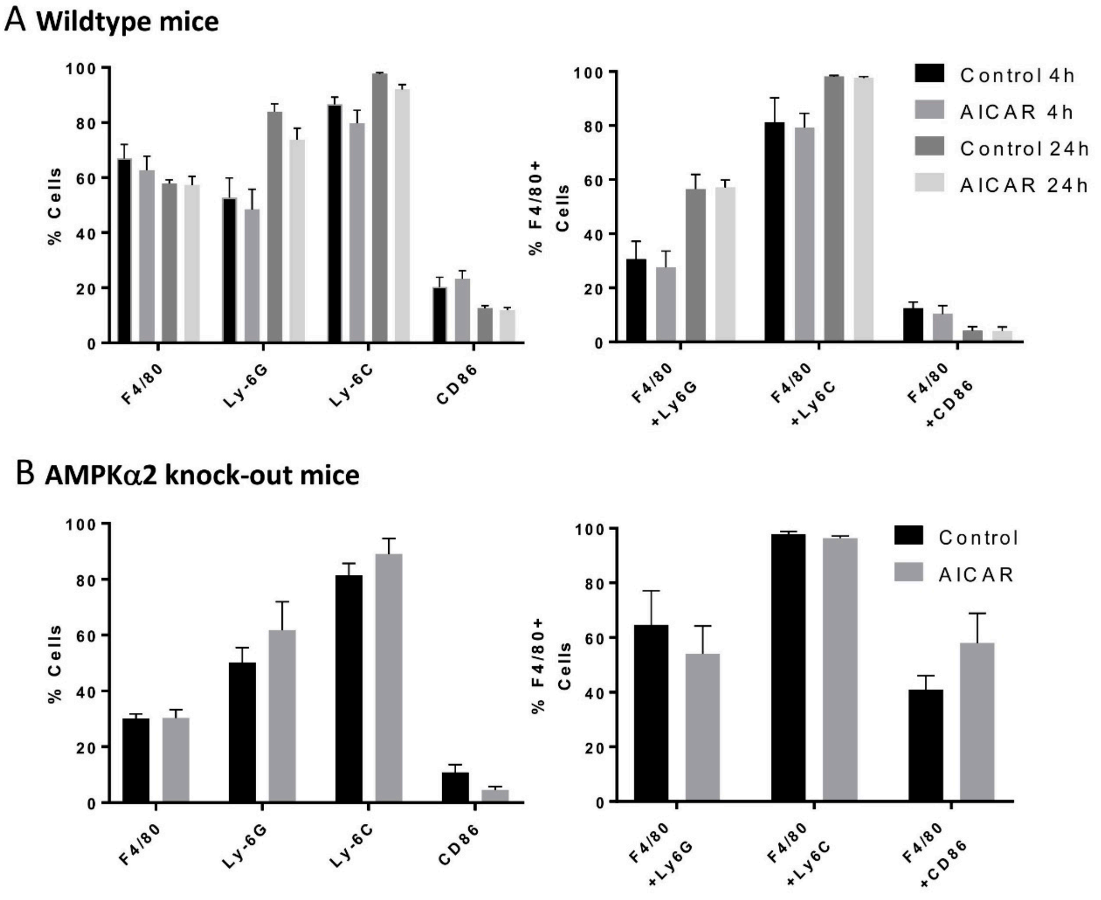

Figure S1. FACS analysis of the composition of immune cells in the zymosan-induced edema. (**A**) FACS analysis of paw tissue of wild type mice 4 and 24h after injection of zymosan A solution into the plantar side of the paw. Left diagram: F4/80+ Ly-6G+ Ly-6C+, and CD86+ cells in the paw. Right diagram: Percentage of Ly-6G+ Ly-6C+, and CD86+ cells in F4/80+ macrophages in control or AICAR-treated mice (n=3-4/group, 4h after zymosan; n=9/group, 24 hours after zymosan). (**B**) FACS analysis of paw tissue of AMPKα2 knock-out mice 24h after zymosan A injection. Left diagram: F4/80+, Ly-6G+, Ly-6C+, and CD86+ cells in the paw, right diagram: Percentage of Ly-6G+, Ly-6C+, and CD86+ cells in F4/80+ macrophages in control or AICAR-treated mice (n=3-4/group). Statistical calculation of these analyses showed no significant differences.

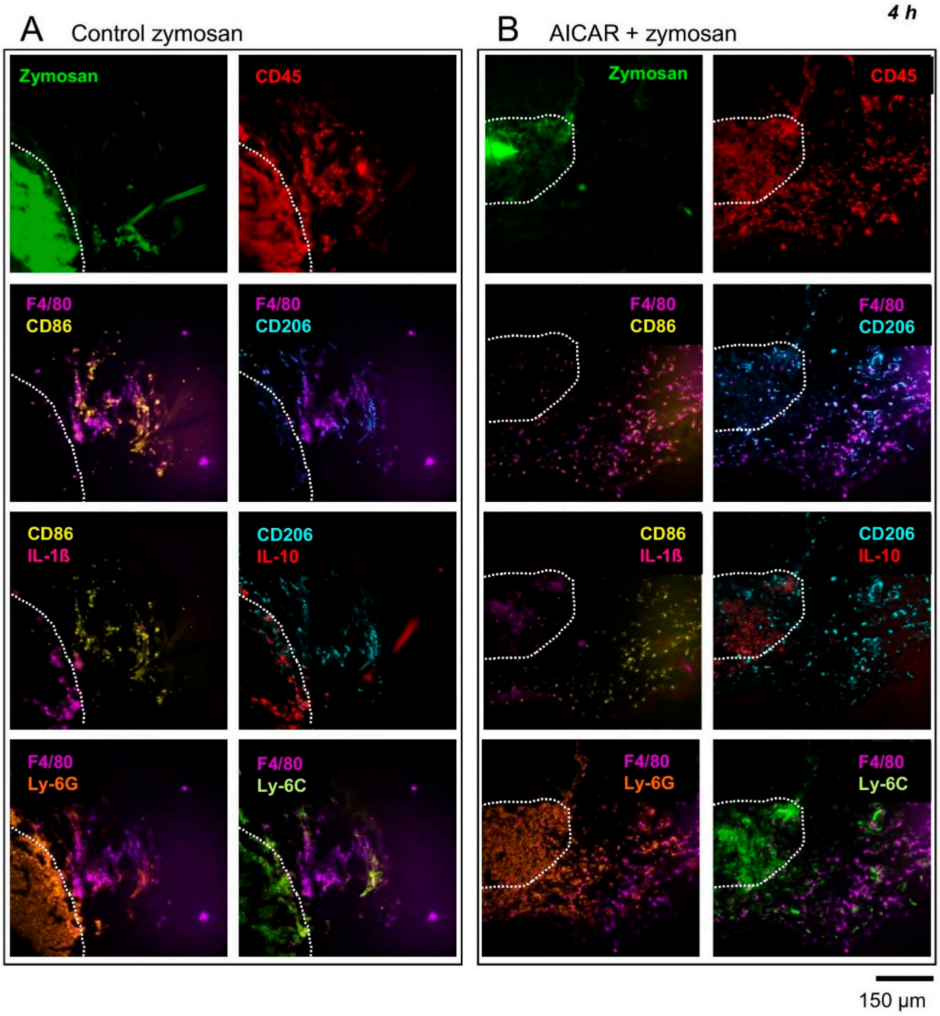

Figure S2. MELC analysis of immune cells and inflammatory markers in the zymosan-induced edema. (**A**) and (**B**) are the representative MELC immunostainings of macrophage markers in the paw of mice 4 h after injection of zymosan (**A**) or zymosan + AICAR (**B**). The dotted white lines indicate the outline of the area containing zymosan as shown in the upper left panel. The respective images show CD45 staining (red) as well as co-stainings of F4-80 (pink) with CD86 (yellow), CD206 (turquoise), Ly-6G (orange), and Ly-6C (light green), respectively. Furthermore, co-stainings are shown for CD86 with IL-1β (pink) and CD206 with IL10 (red). The pictures show representative images of three independent experiments. The images are shown in false colors.

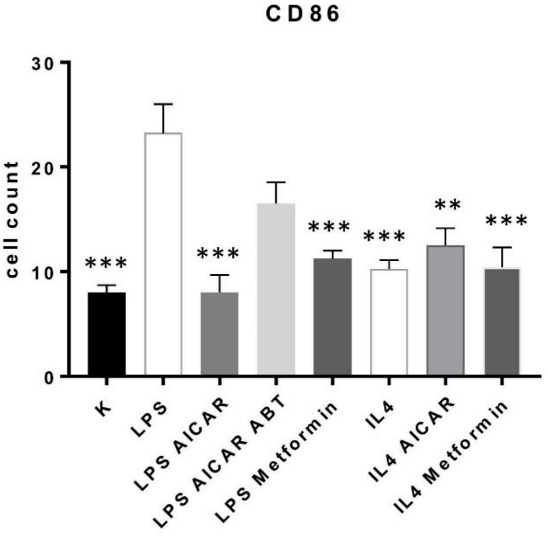

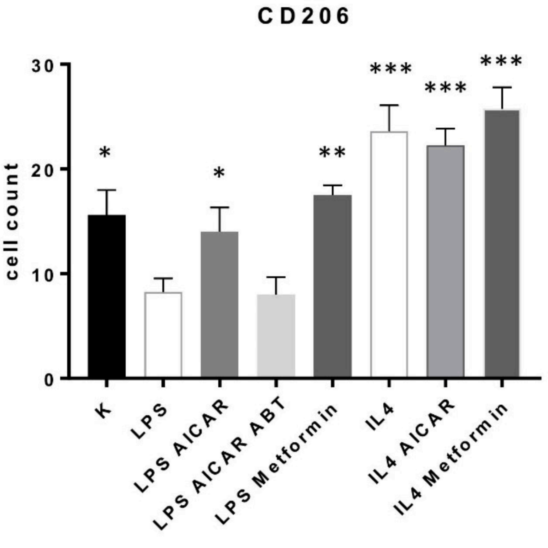

Figure S3. Quantitative analysis of CD86 and CD206 immunofluorescence in bone marrow macrophages. Immunofluorescence stainings of BMM labeled with antibodies against CD86 and CD206 were quantitatively analyzed using Image J software. (n=4/group). *p < 0.05, **p< 0.01, ***p< 0.001 statistically significant difference in comparison with LPS-treated controls.

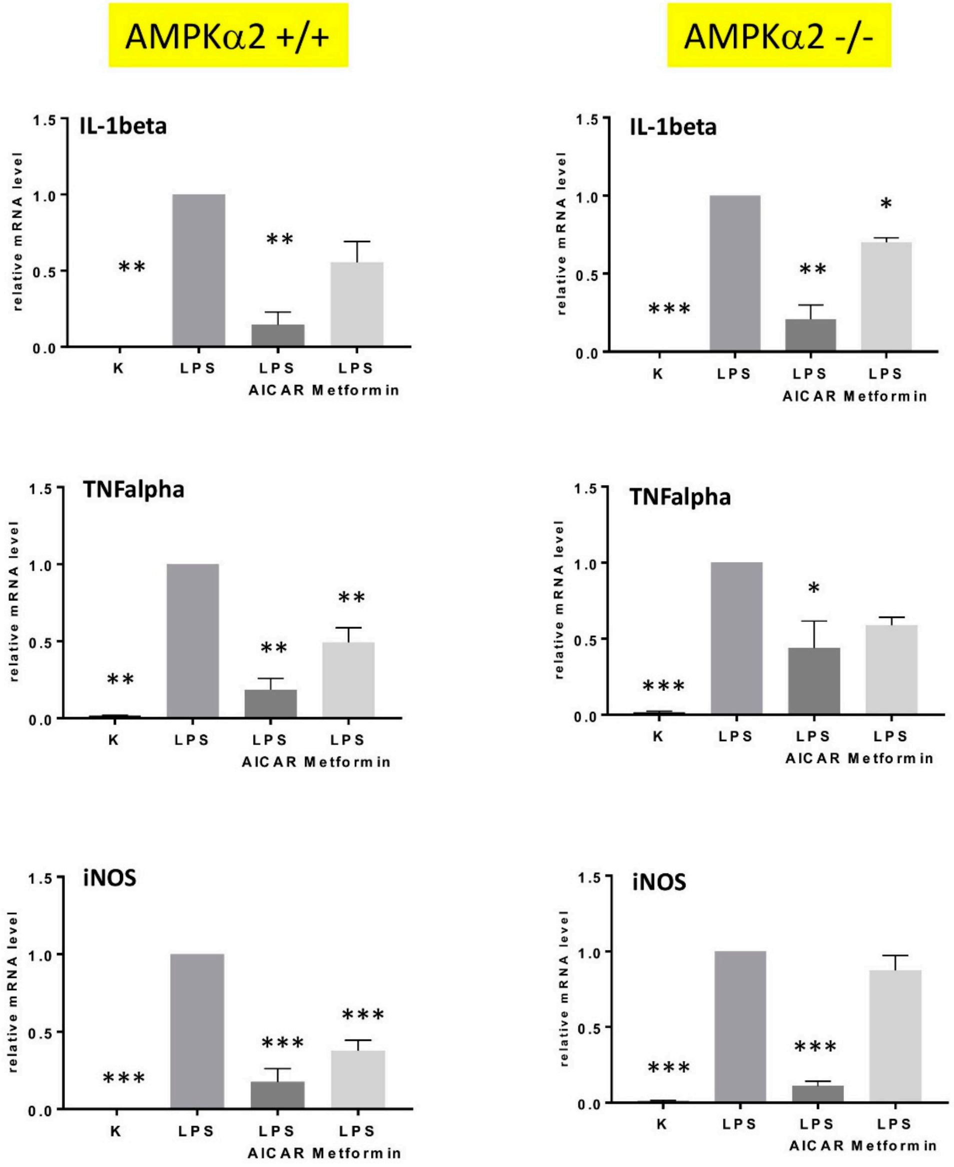

Figure S4. Effects of AICAR and metformin on the expression of inflammatory genes in primary bone marrow derived macrophages derived from AMPKα2 wild type and knock-out mice. RT-PCR analyses of proinflammatory IL1beta, TNF alpha, and iNOS in LPS-stimulated BMMs of AMPKα2 wild type (n=4-5/treatment) and knock-out mice (n=3/treatment). *$p < 0.05$, **$p < 0.01$, ***$p < 0.001$ statistically significant difference in comparison with LPS-treated controls.
